# Non-alcoholic Steatohepatitis Pathogenesis, Diagnosis, and Treatment

**DOI:** 10.3389/fcvm.2021.742382

**Published:** 2021-09-07

**Authors:** Bo Zhu, Siu-Lung Chan, Jack Li, Kathryn Li, Hao Wu, Kui Cui, Hong Chen

**Affiliations:** Department of Surgery, Vascular Biology Program, Harvard Medical School, Boston Children's Hospital, Boston, MA, United States

**Keywords:** non-alcohol fatty liver disease, non-alcoholic steatohepatitis, pathogenesis, diagnosis, treatment

## Abstract

There has been a rise in the prevalence of non-alcohol fatty liver disease (NAFLD) due to the popularity of western diets and sedentary lifestyles. One quarter of NAFLD patients is diagnosed with non-alcoholic steatohepatitis (NASH), with histological evidence not only of fat accumulation in hepatocytes but also of liver cell injury and death due to long-term inflammation. Severe NASH patients have increased risks of cirrhosis and liver cancer. In this review, we discuss the pathogenesis and current methods of diagnosis for NASH, and current status of drug development for this life-threatening liver disease.

## Introduction

There has been an increased interest in non-alcoholic fatty liver disease (NAFLD), and its advanced stage, non-alcoholic steatohepatitis (NASH) because of their increasing impact on global health (1). In the United States, the number of NAFLD cases is rapidly expanding, and is expected to reach 100.9 million patients in 2030 (~1/3 of the population) (2). During the past three decades, the number of patients with NAFLD has increased from 20 to 32% of the United States population (3). Recently, there is an increasing tendency for young people to be diagnosed with NAFLD due to being overweight or obese (4). As an increased proportion (~25% of NAFLD cases) of NAFLD will lead to NASH, there will be increased number of NASH patients with cirrhosis, leading to elevated liver transplantation for end-stage cirrhosis (5). Worse still, the risk of hepatocellular carcinoma (HCC) increases significantly for NAFLD or NASH patients who have cirrhosis (6–8).

NASH is strongly associated with overweight or obesity and metabolic syndromes (9, 10). Recent studies have shown that more than 80% of patients with NASH are overweight or obese (11, 12). NASH is highly associated with type 2 diabetes mellitus (13–15). NAFLD is the general term that comprises hepatic steatosis and steatohepatitis. Unlike isolated hepatic steatosis, NASH is strongly associated with fibrosis found in liver biopsy (5). The level of fibrosis varies among different NASH patients and advanced fibrosis progresses into liver cirrhosis and the eventual scarring (16). Currently, fibrosis-induced liver cell death and further functional failure is a major cause of liver transplantation (17). In addition to liver functional failure, cirrhosis and HCC, associated non-liver adverse outcomes are primarily related to increased cardiovascular diseases (18–21) and type 2 diabetes mellitus (22). Recently, studies have suggested that NAFLD should be defined as a disease of global metabolic dysfunction (23), and not just limited to the liver. The metabolic dysfunctions in fat tissue and muscle, and microbiota variation in the gut contribute to fatty liver disease progression. Therefore, to more accurately describe NAFLD, scientists suggest renaming it as “metabolic associated fatty liver disease” or MAFLD (24).

At present, steady progress in clarifying the pathogenesis of NASH has been made, leading to the identification of therapeutic targets for drug development. However, there is currently no FDA-approved drug that can cure NASH. In addition, the lack of precise predictive biomarkers limits early diagnosis of NASH. Liver biopsy remains the gold standard for diagnosis of NASH. This review focuses on discussing the risks for NASH pathogenesis, current development of biomarkers, and therapeutic target identification for drug development.

## Pathogenesis of NAFL/NASH

**Figure 1:**
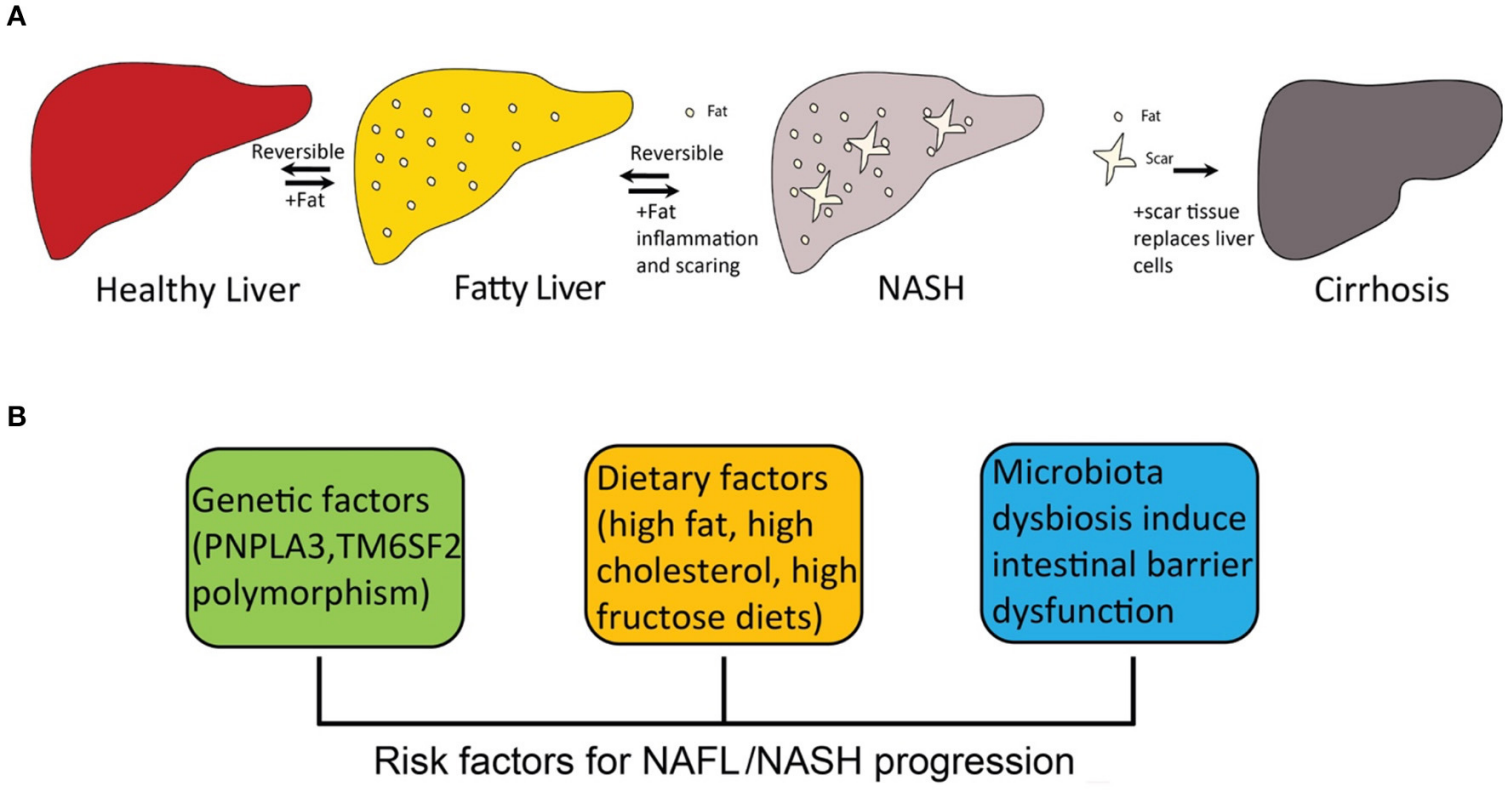
The progression of fatty liver disease and risk factors for NAFL/NASH progression. **(A)** the spectrum of fatty liver disease; **(B)** risk factors for NAFL/NASH progression.

The “two-hit” theory for the development of NASH was first proposed more than two decades ago (25). The theory assumed that the setting in of steatosis is the first hit, and that a second hit from other factors is required for the development of NASH (26), such as oxidative stress. However, this theory is now considered outdated. Multiple-hit pathogenesis was proposed, suggesting that many different factors have been considered to contribute to NASH progression, such as inherited and environmental factors (27, 28). Diet-induced obesity is the most common inducer of NASH development, because the severe accumulation of fat in liver leads to dysfunction of lipid metabolism. At present, the accumulation of hepatic free cholesterol and free fatty acid is considered the primary source of stress to the liver (29–31). Particularly, hepatic free cholesterol is a major lipotoxic molecule critical for NASH progression (32). Its metabolites trigger hepatocellular stress (for example, oxidative stress) and induce hepatocyte injury and death, leading to fibrosis and further cirrhosis (Figure 1A).

Fibrosis in the liver and hepatocyte injury and death are the key features that distinguish NASH from isolated steatosis (5, 33). It is, however, debatable whether hepatocyte injury causes liver inflammation or if hepatocyte injury is the consequence of liver inflammation. Both hepatocyte injury and liver inflammation are relevant to the pathogenesis of NASH because injured hepatocytes release factors that promote inflammation, resulting in a downward spiral as inflammation further triggers hepatocyte injury (34, 35). This is because inflammation in the liver is caused by released proinflammatory chemokines and cytokines (such as TNF-α, IL-6, and CCL2), which further damage the injured hepatocytes (36). In the liver, Kupffer cells, a kind of resident macrophage, are localized in the lumen of the liver sinusoids and play a central role in liver inflammation (37). The initiation of liver injury stems from the activation of Kupffer cells resulting in cytokine and chemokine production (38). Kupffer cells can be divided into classically activated M1 Kupffer cells (proinflammatory M1) and alternatively activated M2 Kupffer cells (wound-healing M2) (39). The activation of pro-inflammatory M1 Kupffer cells is the critical step that contributes to the pathogenesis of fibrogenesis during NASH progression. In contrast, the polarization and activation of anti-inflammatory M2 Kupffer cells play a protective role against fibrogenesis in NASH (40). Importantly, M2 Kupffer cells promote the apoptosis of M1 Kupffer cells, which is protective against NAFL/NASH (41). Under high fat diet treatment, mice with a high M2:M1 Kupffer cell ratio are resistant to developing liver lesions, while mice with a high M1:M2 Kupffer cell ratio are more likely to develop liver lesions (42).

In addition to resident Kupffer cells in liver, monocyte-derived macrophages play an important role in the pathogenesis of NAFL/NASH (40). The infiltrating monocyte-derived macrophages can be divided into two major subtypes, Ly-6C^hi^ macrophages and Ly-6C^lo^ macrophages (43), both of which affect hepatic stellate cells (HSCs), though in differing ways. HSCs have been identified as the major extracellular matrix protein (ECM) producing cells in injured liver (44) that play the central role in the formation of hepatic fibrosis (45). HSCs have two different states, quiescent and activated states, and the transdifferentiation from the quiescent into the activated state is the major cause of fibrosis (44). The proinflammatory Ly-6C^hi^ macrophages activate HSCs by secreting IL-1β and CCL2 that enhance the fibrotic process (46), while the pro-restorative Ly-6C^lo^ macrophages promote apoptosis of HSCs and accelerate extracellular matrix degradation by upregulation of matrix metalloproteinase 9 (MMP9), MMP12 and MMP13 (47). The pro-restorative Ly-6C^lo^ macrophages express chemokine (C-X3-C motif) receptor 1 (CX3CR1), and because its ligand1 (CX3CL1) is mainly expressed in HSCs, CX3CL1-CX3CR1 interaction negatively regulates inflammatory properties in macrophages within the liver (48). Chemokine CCL2 production, working through CCR2, is a major cause of monocyte-derived macrophage (inflammatory Ly-6C^hi^ macrophages) recruitment induced by Toll-like receptor 4 (TLR4) signaling (49). The infiltration of monocytes from blood that rapidly differentiate into pro-inflammatory macrophages in the liver contributes to NAFL/NASH progression (46). Extensive studies have reported that genetic deficiency or pharmacological inhibition of CCR2 decreased monocyte recruitment to the liver and ameliorated NASH in mice (50–52). In addition to chemokine CCL2, cytokines like TNF-α and IL-1β released from macrophages are important drivers of steatosis, inflammation, and fibrosis in NAFL/NASH (40).

A number of risk factors promote the pathogenesis of NASH, including inherited and environmental factors (Figure 1B). Dietary factors are one of the most important environmental factors that lead to NASH.

### Genetic and Epigenetic Factors

Genome-wide association studies (GWAS) suggest that polymorphisms in patatin-like phospholipase domain-containing 3 (PNPLA3) and transmembrane 6 superfamily, member 2 (TM6SF2) promote the development of NASH (53). Particularly, PNPLA3 polymorphisms (I148M) are strongly associated with hepatic steatosis caused by accumulation of PNPLA3 on hepatic lipid droplets (54–56). TM6SF2 polymorphism reduces very-low density lipoprotein (VLDL) secretion that is associated with NASH and fibrosis (57, 58). The polymorphism of several other genes, including glucokinase regulator (GCKR), membrane bound O-acyltransferase domain-containing 7 (MBOAT7) and hydroxysteroid 17β-dehydrogenase (HSD17B13), are closely associated with susceptibility to NAFL and progression of NASH (53).

Epigenetic mechanisms are special genetic regulations without any change in gene sequence in the genome but with different modifications, such as DNA methylation, histone modification, and non-coding RNAs (59). By analysis of liver samples from NAFLD patients, the heavy methylation of NAFLD associated genes, such as PNPLA3 (60) and PPARG (61), increases the severity of NAFLD. Recently studies have indicated that non-coding RNAs could regulate NAFLD progression, including miR-122 and miR-125b, which show significantly decreased expression in NAFLD patients (62, 63). Moreover, hepatic-specific deletion of miR-21 prevents steatosis, and thus may be a potential therapeutic target for NAFLD (64). In addition, changes in hepatic lncRNA expression patterns are associated with NAFLD. For example, a strong increase in hepatic lncRNA un.372 is detected in NAFLD patients (65), while hepatic lncRNA lnc18q22.2 is reported to correlate with the severity of NASH (66). A list of microRNAs and long non-coding RNAs that are involved in NAFL/NASH progression are summarized in Table 1. Furthermore, studies in rodents have demonstrated the existence of epigenetic factors that regulate fibrogenic liver cell development to cirrhosis (124). A similar phenomenon is observed in NASH patients. Therefore, epigenetic mechanisms may be related to susceptibility for NASH.

**Table 1:** microRNAs and long non-coding RNAs that are involved in NAFLD pathogenesis.

**miRNAs**	**Expression in NAFL/NASH**	**References**	**lncRNAs**	**Expression in NAFL/NASH**	**References**
miR-15b	Upregulated	(67)	ApoA4-AS	Upregulated	(68)
miR-16	Upregulated	(69)	APTR	Upregulated	(70)
miR-19	Upregulated	(71)	FLRL2	Downregulated	(72)
miR-21	Upregulated	(73)	Gm15622	Upregulated	(74)
miR-22	Upregulated	(75)	HOTAIR	Upregulated	(76)
miR-26a	Downregulated	(77)	HULC	Upregulated	(78)
miR-27a/b	Upregulated	(79)	lncARSR	Upregulated	(80)
miR-29a	Downregulated	(81)	AK012226	Upregulated	(82)
miR-30c	Downregulated	(83)	lnc-HC	Downregulated	(84)
miR-33a/b	Upregulated	(85)	lncHR1	Downregulated	(86)
miR-34a	Upregulated	(87)	Inc-H19	Upregulated	(88)
miR-99a	Downregulated	(89)	lnc-JAM2-6	rs2829145 A/G	(90)
miR-122	Upregulated	(62)	lncLSTR	Downregulated	(91)
miR-125b	Upregulated	(63)	lncRNA Blnc1	Upregulated	(92)
miR-130a	Downregulated	(93)	lncSHGL	Downregulated	(94)
miR-135a	Downregulated	(95)	lnc18q22.2	Upregulated	(66)
miR-146a	Downregulated	(96)	LFAR1	Upregulated	(97)
miR-155	Downregulated	(98)	MALAT1	Upregulated	(99)
miR-181b	Upregulated	(100)	MEG3	Downregulated	(101)
miR-190b	Upregulated	(102)	Mirt2	Downregulated	(103)
miR-192	Upregulated	(104)	MRAK052686	Downregulated	(105)
miR-194	Upregulated	(106)	NEAT1	Upregulated	(107)
miR-197	downregulated	(89)	NONMMUG027912	downregulated	(108)
miR-199a	Upregulated	(109)	NONMMUT010685	Downregulated	(110)
miR-200a/b	Upregulated	(111)	NONMMUT050689	Downregulated	(110)
miR-205	Upregulated	(112)	NR002155.1	Downregulated	(113)
miR-221/222	Upregulated	(114)	PVT1	Upregulated	(115)
miR-223	Upregulated	(116)	RP11-128N14.5	Upregulated	(117)
miR-335	Upregulated	(118)	Runx1	Upregulated	(119)
miR-375	upregulated	(120)	SRA	upregulated	(121)
miR-378	Upregulated	(122)	TGFB2-OT1	Upregulated	(117)
miR-451a	Downregulated	(123)	uc.372	Upregulated	(65)

### Dietary Factors

Consumption of a high-calorie diet with high fat and high sugar (western diet) results in weight gain and is the initial event for the development of fatty liver (125–127). Particular types of lipids and carbohydrates play important roles in the progression of NASH. For dietary lipids, polyunsaturated fatty acids (PUFAs) induce inflammation and fibrosis formation in NASH (128). For dietary carbohydrates, over-consumption of carbohydrates extensively promotes the development of NAFLD (129), especially fructose, a highly lipogenic sugar and a common component in almost all major sweet foods (130, 131). Extensive experimental studies support the association between the increasing rates of obesity and the progression of NAFLD (132–134). Obese individuals who have an excessive body mass index (BMI) and visceral obesity are at a high risk for developing NAFLD (135). Studies have reported that more than 95% people that had severe obesity have NAFLD (136). Type 2 diabetes mellitus (T2DM) patients have higher risks for NAFLD due to insulin resistance, and T2DM and NAFLD can be developed simultaneously (137). In addition, people who have dyslipidemia and hypertension are also at high risk for NAFLD (138).

### Microbiota Dysbiosis

The relationship between intestinal microbiota and NAFL/NASH has been proposed for decades. Intestinal microbiota is altered in genetically obese mice that have metabolic syndrome and fatty liver (139). Studies indicate that microbiota dysbiosis is associated with inflammatory signaling, which promotes hepatic steatosis and NASH (140, 141). Recently, extensive studies on the gut-liver axis have suggested that intestinal microbiota influenced host susceptibility to obesity, hepatic steatosis, and NASH (142–144). The homeostasis of intestinal microbiota is essential to maintaining proper function of the intestinal barrier, and recent studies have shown that intestinal microbiota dysbiosis triggers intestinal inflammation and further impairs the intestinal barrier. Microbial products released can reach the liver and induce hepatic inflammation and further lead to NAFL/NASH (145). This intestinal barrier is hepatoprotective, and microbiota-driven gut-vascular barrier disruption is a prerequisite for NASH development (146). This barrier disruption is caused by diet-induced dysbiosis (147). Patients with NAFL and NASH usually show dysbiosis in the gut microbiota (148). Overall, patients with NAFL and NASH have lower microbiota diversity than healthy people and have different microbiota species abundance patterns (149). For example, patients with NASH have an increased abundance of *Escherichia*, where patients with advanced fibrotic NASH or cirrhosis have dramatically increased proportions of *Bacteroides* and *Ruminococcus* (150).

## Diagnosis of NAFL/NASH

**Figure 2:**
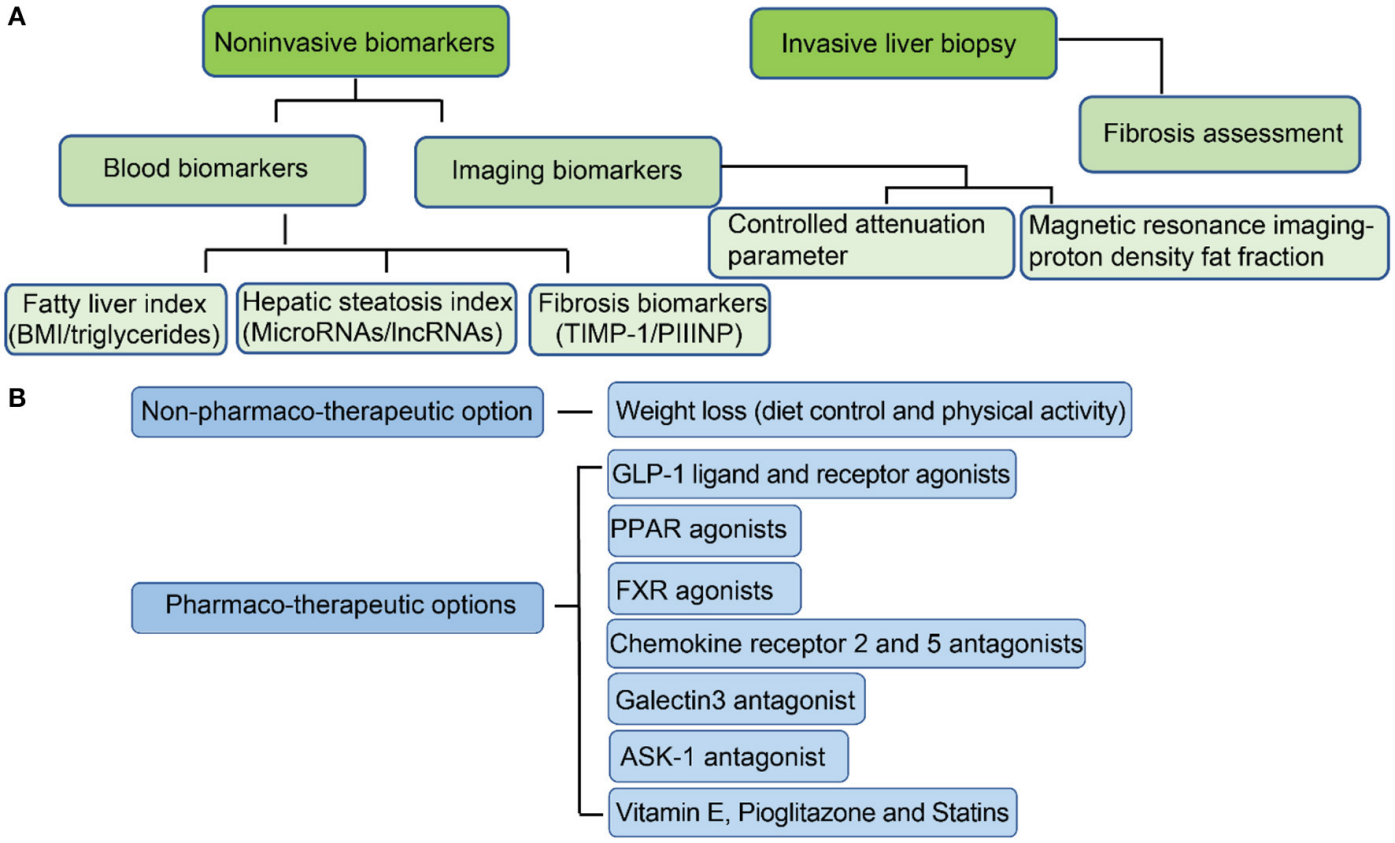
Current approaches for the diagnosis of NAFL/NASH and options for treatment. **(A)** Methods for NAFL/NASH diagnosis; **(B)** Options for NAFL/NASH treatment.

At present, liver biopsy remains the gold standard for diagnosis of (151), since invasive liver biopsy for assessing different fibrosis stages is the most accurate method for NASH diagnosis (Figure 2A) (152). However, liver biopsy is painful for patients, and has sampling error limitations (153). Therefore, new developments in non-invasive biomarkers for early diagnosis and treatment of NAFL and NASH are urgently needed. Current progression on developing non-invasive biomarkers is mainly based on the detection of hepatic steatosis (154). There are several indices and scores for the evaluation of hepatic steatosis. Testing of blood biomarkers, determining the fatty liver index (FLI) and determining the hepatic steatosis index (HSI) are the most popular methods to evaluate the risks of NAFLD (155). Fatty liver index, BMI, and serum level of triglycerides also have moderate accuracy for fatty liver diagnosis (156). Hepatic steatosis index can be evaluated by the ratio between serum aspartate aminotransferase (AST) and alanine aminotransferase (ALT) and has moderate accuracy for the detection of fatty liver (156). The limitation of both FLI and HSI is insensitive to mild steatosis since they are designed to target indirectly on blood fat rather than liver fat.

A more sensitive and accurate method is to measure liver fat directly. The development of imaging biomarkers has dramatically improved the progress of NAFLD diagnosis (157). Ultrasonography is the most common method for detection of hepatic steatosis and can accurately identify moderate to severe steatosis (158). However, it is less sensitive when detecting steatosis in NASH patients who have advanced fibrosis (159). Computed tomography (CT), although sensitive in detecting moderate and severe steatosis with mild histological fibrosis (159), has not been shown to have improved sensitivity in mild steatosis (160). Both controlled attenuation parameter (CAP) and magnetic resonance imaging-proton density fat fraction (MRI-PDFF) can address sensitivity issues in mild steatosis (161, 162). Controlled attenuation parameter is a method for grading steatosis that measures the degree of ultrasound attenuation by hepatic fat using a process based on simultaneous transient elastography, which is more sensitive and accurate than previous imaging markers (163). MRI-PDFF maps can be generated within seconds and detection of hepatic fat is more accurate than CAP for detecting all grades of steatosis in patients with NAFLD (164). Magnetic resonance imaging (MRI) approaches 100% sensitivity in the detection of hepatic steatosis, even at low steatosis levels (165). At present, MRI is the best method for detecting hepatic steatosis accurately and efficiently due to its high sensitivity.

The current progress on non-invasive biomarkers development for fibrosis diagnosis has advanced. Several potential non-invasive biomarkers have been reported for NASH diagnosis. Studies indicate that serum hyaluronic acid (HA) is a non-invasive marker of liver fibrosis (166), and serum YKL-40 can also be a marker of liver fibrosis in patients with NAFLD (167). In addition, the expression of tissue inhibitor of matrix metalloproteinase 1 (TIMP-1) is increased in human and rat models of liver fibrosis (168), and its degree of expression correlates with the extent of fibrosis in human liver (169). Although functional studies indicate TIMP-1 promotes hepatic fibrogenesis, TIMP-1 deficiency does not prevent carbon tetrachloride (CCl4)-induced hepatic fibrogenesis (170). Terminal peptide of procollagen III (PIIINP) is released during the synthesis and deposition of type III collagen (171). At present, PIIINP has been validated to be effective in the detection of fibrosis, and is particularly effective in the detection of severe fibrosis (172). PIIINP is elevated in patients with advanced fibrosis (172). Therefore, serum PIIINP is potentially a good non-invasive marker of liver fibrosis.

## Treatment of NAFL/NASH

Most NASH patients are obese. The first and simplest management against NAFLD or NASH is to make lifestyle modifications (173). Through sustained weight loss (such as through a calorie-restricted diet) and increased physical activity (exercise), hepatic steatosis caused by western-style diet and sedentary lifestyle can be reduced significantly (174). A modest weight loss of about 3% may reduce hepatic steatosis, however, up to 10% or more is required for the resolution of NAFLD and the regression of fibrosis in NASH (175). Thus, resolution of steatosis in NAFLD and even fibrosis reversal in NASH can be achieved after significant weight loss through lifestyle modifications. Therefore, as a non-pharmaco-therapeutic option, transitioning from a high-calorie diet and a sedentary lifestyle into a restricted calorie diet with increased physical activity is the healthiest treatment for NAFL and early stage of NASH.

In addition to lifestyle modifications, there are several therapeutic targets that are under clinical trials (Figure 2B). The most promising pharmaco-therapeutic candidates will be discussed.

### Vitamin E, Pioglitazone, and Statins

Oxidative stress plays an essential role in the development from isolated steatosis to NASH (176). Anti-oxidation treats NAFL/NASH by removing excessive reactive oxygen species (ROS) (177). Extensive studies have indicated that vitamin E reduced steatosis and ameliorated fibrosis (178–180). However, for NASH patients with diabetes, vitamin E alone does not significantly improve histological outcomes (181). Considering NAFL/NASH is a metabolic syndrome, and thus is usually accompanied by other metabolic diseases, such as diabetes and CVD, drugs for the treatment of diabetes and CVD are used for treating NAFL/NASH, particularly pioglitazone and statins (182, 183). Pioglitazone is used for treatment of type 2 diabetes mellitus, and has been reported to significantly reduce fibrosis in NASH patients (184). The safety and efficacy of low-dose pioglitazone (NCT04501406) has been tested (185). In addition, statins, a class of drugs used for reducing cardiovascular disease risk, has been tested on NAFL/NASH patients who then displayed substantial improvement on steatosis and fibrosis (186, 187). While piloglitazone and statins have already been in the market for a while for treating other conditions, they also represent new potential therapies for NAFL/NASH.

### GLP-1 Ligand and Receptor Agonists

Glucagon-like peptide 1 (GLP-1) is essential for glucose homeostasis (188). GLP-1 and its receptor agonists (GLP-1 RAs) can treat type 2 diabetes mellitus (189), which is closely associated with NAFLD (13). Evidence supports that GLP-1 secretion is impaired in patients with NAFL and NASH (190), indicating that GLP-1 agonists may be potential treatment candidates for NAFLD. GLP-1 agonists and GLP-1 RAs, including liraglutide (NCT01237119), exenatide (NCT00650546) and semaglutide (NCT02970942), have been tested for improving liver histology in NASH patients (191–193), and are currently under phase II clinical trials for NASH.

### PPAR Agonists

Peroxisome proliferator-activator receptors (PPARs) are a group of nuclear receptors that play a critical role in intracellular lipid metabolism (194). Extensive studies indicate the dual PPAR alpha and delta agonist, elafibranor, is effective against NASH (195). Taking elafibranor at a dose of 120 mg/day has been shown to cause significant regression of fibrosis in NASH patients. In addition, other metabolic parameters, such as liver enzymes, are also greatly improved by elafibranor. Elafibranor is now under phase III clinical trials (NCT02704403).

### FXR Agonists

Farnesoid X receptor (FXR) is a master regulator of hepatic triglyceride and glucose homeostasis (196). Obeticholic acid (OCA), a FXR agonist, has been recently studied and shown to improve histology and fibrosis scores (197). OCA is highly promising for NASH treatment and is under phase III clinical trials (NCT02548351). In addition to OCA, other FXR agonists are also under clinical trials for treating NASH. Another FXR agonist, MET-409, significantly reduces liver fat after 12 weeks of treatment in NASH patients (198). MET-409 is under phase II clinical trials for NASH (NCT04702490). EDP-305, a potent FXR agonist, reduces liver fat and is a potent inhibitor of fibrosis (199). EDP-305 is under phase II clinical trials for NASH (NCT03421413).

### CCR2/5 Antagonists

NASH is a disease with inflammation and fibrosis. Chemokine receptor 2/5 (CCR2 and CCR5) are commonly increased in the liver from NASH patients. Cenicriviroc (CVC), a C-C motif chemokine receptor 2/5 (CCR2/5) antagonist, was developed to target inflammation (200). In NASH patients, liver fibrosis is caused by the accumulation of extracellular matrix proteins, mainly composed of collagen (201). CCR5 antagonist can inhibit collagen production by HSCs by impairing the migration, activation, and proliferation of HSCs (202). CVC has been shown to have antifibrotic function and has significantly improved fibrosis in NASH patients after 1 year of treatment (203). CVC is currently under phase III clinical evaluation for NASH (NCT03028740).

### Galectin-3 Antagonist

Galectin-3 protein expression is essential for the development of hepatic fibrosis, which is significantly increased in NASH liver (204). In mice models, GR-MD-02, a galectin-3 inhibitor, markedly ameliorates liver fibrosis through inhibition of collagen deposition (205). GR-MD-02 has been shown to be safe and efficient for patients with NASH cirrhosis with portal hypertension (165). The safety and efficacy of GR-MD-02 for the treatment of liver fibrosis is under phase II clinical trials (NCT02462967) (206).

### ASK-1 Antagonist

To improve inflammation and fibrosis in NASH, many therapeutic targets have been tested. The inhibitors of apoptosis signal-regulating kinase 1 (ASK1), a serine/threonine kinase, have been shown to significantly improve fibrosis in NASH animal models (207). Selonsertib (aka GS-4997), a selective inhibitor of ASK1, can reduce hepatic steatosis and fibrosis in NASH animal models fed with high fat and sugar. In phase II clinical trials, patients treated with selonsertib showed huge improvements in fibrosis through reduction of hepatic collagen content (208). However, a recent study has indicated that selonsertib neither leads to fibrosis regression nor reduces NASH progression (209). At present, selonsertib is undergoing phase III clinical evaluation and being studied for its efficacy on NASH (NCT03053050).

### Gut and Microbiome Related Therapies

Studies between microbiome and NAFL/NASH are relatively nascent, but the consensus is that dysbiosis leads to increased intestinal permeability, which may increase NAFL/NASH progression (210). Thus, disturbed gut-liver barrier integrity is essential in the pathogenesis of both NAFL and NASH since the release of bacterial products from the gut into blood circulation may cause a massive inflammatory response from the liver (211). Orlistat, an FDA-approved lipase inhibitor for treating obesity, reduces absorption of dietary fat (212) and may have beneficial effects on body weight through modification of the composition of gut microbiota (213), however, the efficacy of orlistat on NASH has not been clearly demonstrated. Other drugs targeting the microbiome include solithromycin. Solithromycin is an antibiotic in clinical trials for the treatment of bacterial infection. Studies in a mouse model of NASH have displayed that solithromycin had beneficial effects by reducing hepatocyte ballooning and inflammation (214). Solithromycin is currently in a phase II trial (NCT02510599) for NASH. All six patients with NASH it was tested on showed reduction in NASH parameters after 90 days of treatment (215). Notably, solithromycin may not impair the gut microenvironment since its mechanism of action against NASH may not be related to its antibacterial activity, as it is not active against gut Gram-negative bacteria (214).

Although many potential candidates for the treatment of NASH are under phase II and III clinical trials as discussed above, no drug has yet been approved by the FDA. In addition to the discovery of novel promising targets for clinical testing, existing drugs like piloglitazone and statins used for the treatment of diabetes and CVD diseases can also be viable therapeutic options for NAFL/NASH. Furthermore, antioxidant vitamin E can be a beneficial supplement for NAFL/NASH patients.

## Future Perspectives

Considering NAFLD is closely associated with metabolic dysfunction, metabolic dysfunction-associated fatty liver disease (MAFLD) may better describe the disease than NAFLD (216). The lack of appropriate NASH animal models is the bottleneck for NASH investigation. Although a methionine-choline-deficient (MCD) diet is a widely used model for NASH study, the body weight loss and increased insulin sensitivity of this model are not features of NASH (217). Recently, high fat, high fructose, high cholesterol diets and use of chemical inducers (such as carbon tetrachloride, CCl4) have been extensively applied as NASH models, though the feeding or induction cycle is very long (218). Despite its long-term feeding cycle, this animal model may become more popular in the future since it can accurately mimic the features of NASH.

Non-invasive diagnosis approaches can primarily detect hepatic steatosis sensitively but struggle to detect fibrosis, meaning the diagnosis of NASH still requires invasive liver biopsy. Development of non-invasive methods that can sensitively and accurately diagnose NASH is currently ongoing and such methods may be available in the future. At present, because of the absence of any existing FDA-approved medication for the treatment of NASH, an effective clinical pharmacotherapy is urgently needed for advanced NASH. For mild NASH, the most healthy and effective treatment is through management of diet and lifestyle. NASH fibrosis regression can occur with 10% weight loss, but such weight loss is difficult to achieve. In addition, NASH is commonly associated with diabetes, meaning drugs that target diabetes may have potential for treating NASH. In addition, the development of drug targets on gut microbiota may be a novel direction in the coming decades, because accumulating evidence shows microbiota dysbiosis is one of the critical causes of NASH through the gut-liver axis. Alternatively, the development of pharmacological interventions targeting the polarization of M2 Kupffer cells during the early stages of NASH may become an attractive strategy for reducing inflammation and hepatocyte injury. Similarly, in monocyte-derived macrophages, it is attractive to develop pharmacological interventions that target the polarization of pro-restorative Ly-6C^lo^ macrophages to treat NASH.

## Conclusion

Great developments in NASH pathogenesis, diagnosis and treatment have been achieved in the past decades. This review discusses factors that may induce NASH, non-invasive methods for NASH diagnosis, and potential pharmaco-therapeutic options to resolve NASH. Dietary factors are a major cause for NAFLD that may further develop into NASH, particularly for people under long-term high fat and high sugar consumption who are obese or overweight. Body weight loss is beneficial for early-stage NASH. Accurate and sensitive non-invasive diagnosis methods for NASH are needed to replace invasive liver biopsy. Although no FDA-approved drug is available for NASH, many clinical pharmacotherapies are under phase II or phase III clinical trials. Moving forward, more potential NASH targets will likely be identified, offering more opportunities to discover effective and specific drugs to treat and resolve NASH.

## Author Contributions

BZ, S-LC, and HC wrote the manuscript. JL and KL edited the manuscript. HW and KC critically read the manuscript. All authors contributed to the article and approved the submitted version.

## Funding

This work was supported in part by NIH Grants Nos. R01HL093242, R01HL137229, R01HL133216, and R01HL156362 to HC.

## Conflict of Interest

The authors declare that the research was conducted in the absence of any commercial or financial relationships that could be construed as a potential conflict of interest.

## Publisher's Note

All claims expressed in this article are solely those of the authors and do not necessarily represent those of their affiliated organizations, or those of the publisher, the editors and the reviewers. Any product that may be evaluated in this article, or claim that may be made by its manufacturer, is not guaranteed or endorsed by the publisher.
